# Melody complexity of infants’ cry and non-cry vocalisations increases across the first six months

**DOI:** 10.1038/s41598-021-83564-8

**Published:** 2021-02-18

**Authors:** Kathleen Wermke, Michael P. Robb, Philip J. Schluter

**Affiliations:** 1 Center for Pre-Speech Development & Developmental Disorders, University Hospital, University of Würzburg, Pleicherwall 2, 97070 Würzburg, Germany; 2 Department of Communication Sciences and Disorders, Pennsylvania State University, State College, USA; 3 School of Health Sciences, University of Canterbury - Te Whare Wānanga O Waitaha, Christchurch, New Zealand; 4 School of Clinical Medicine, Primary Care Clinical Unit, The University of Queensland, Brisbane, Australia

**Keywords:** Evolution, Psychology

## Abstract

In early infancy, melody provides the most salient prosodic element for language acquisition and there is huge evidence for infants’ precocious aptitudes for musical and speech melody perception. Yet, a lack of knowledge remains with respect to melody patterns of infants’ vocalisations. In a search for developmental regularities of cry and non-cry vocalisations and for building blocks of prosody (intonation) over the first 6 months of life, more than 67,500 melodies (fundamental frequency contours) of 277 healthy infants from monolingual German families were quantitatively analysed. Based on objective criteria, vocalisations with well-identifiable melodies were grouped into those exhibiting a simple (single-arc) or complex (multiple-arc) melody pattern. Longitudinal analysis using fractional polynomial multi-level mixed effects logistic regression models were applied to these patterns. A significant age (but not sex) dependent developmental pattern towards more complexity was demonstrated in both vocalisation types over the observation period. The theoretical concept of melody development (MD-Model) contends that melody complexification is an important building block on the path towards language. Recognition of this developmental process will considerably improve not only our understanding of early preparatory processes for language acquisition, but most importantly also allow for the creation of clinically robust risk markers for developmental language disorders.

## Introduction

The ability to perceive and produce the time varying vocal fundamental frequency (fo) (i.e., melody) is an extremely important component of auditory information and an essential suprasegmental aspect of spoken language. Each language is characterized by specific musical elements in the form of prosody, that is, its intonation system and constituent rhythm^1^. Human adults^2^ and even newborns are able to distinguish different languages using prosodic cues^3–6^, particularly melody (intonation). Most, if not all, of the linguistic and paralinguistic functions of intonation systems seem to be shared by languages of even widely different origins, a fact which strongly points to their universal role for language acquisition^1^.

Infants recognize auditory information of the surrounding language on the basis of prosodic cues, mainly melody, the prototypical musical element of language^7–14^. They do so long before they are capable of perceiving the segmental characteristics of speech such as consonants, vowels, or syllables. Infants apply their knowledge of melody in order to segment the continuous speech stream into meaningful parts (prosodic phrasing)^15,16^ and experience how protowords begin to emerge from the melody^17^.

Melody contour is probably the most important entity to be memorized and imitated by infants^13,18–22^. A newborn’s cortical structures are active in the processing of prosodic information, particularly melody contour, as typified in the exaggerated melodic expression of infant-directed speech (IDS)^23,24^. IDS exemplifies how messages are conveyed to the preverbal human infant by melody variation^18,20,25–27^. According to Falk^28,29^, the special, melodic form of vocal communication that exists between a mother and her infant (IDS) could well have co-evolved in prehistoric time. The essential characteristic of IDS is the emotional content conveyed via melody, rather than words. The well-developed perception of melody and rhythm in the foetus^30^, newborn^31–33^ and young infant^21,34–37^ leaves no doubt that the human infant has a specific sensitivity to musical sound features^36,38–40^.

During the first two months, vocal messages are almost entirely and very effectively coded in the laryngeally produced cry melody, while supralaryngeal mechanisms are still immature^41,42^. In addition to crying, from about the end of the second month of life onwards, new sound properties emerge within the non-cry vocal repertoire of infants. These properties (e.g., consonant- and vowel-like elements)^42,43^, are the result of maturing supralaryngeal mechanisms that often change the sound to be recognized as seemingly more “speech-like”. In most of these non-cry vocalisations, melody still serves as a kind of scaffolding and forms together with supralaryngeally produced constituents the characteristic overall shape (gestalt) of the sound^17,44,45^.

Given the importance of melody for language acquisition, there remains a dramatic lack of knowledge with respect to the starting point of melodic variation and age-related development of melody patterns in infants’ cry and non-cry vocalisations. Commonly used models of vocal development describe the emerging speech capacity by focussing on the occurrence of segmental (syllable-like) precursors, sometimes called “protophones” after Oller^46–48^. However, the specific protophone typology of pre-speech sounds is lacking the crucial importance of prosodic development and hence, does not fully conform with or capture a young infant’s entire vocal behavior^49^.

Considerable importance is placed on the fo patterns of non-cry vocalisations within the field of pre-speech development and language acquisition^10,42,49–54^. In contrast to melody in non-cry vocalisations, cry melody is often ignored in models of vocal development towards language^47,55^. The first universal step towards prosody (intonation and rhythm), and hence language development for the young infant requires coordination of respiratory and laryngeal activity for the production of melody variations (phonation)^56,57^. There is compelling evidence for a huge melody repertoire in infants’ spontaneous natural crying, recorded in a relatively ‘relaxed context’ in the presence of the mother (i.e., when the infants were hungry or thirsty)^45,58–60^. This is completely different to the fo pattern of cries elicited by application of a painful stimulus. The medical importance of vocal fo and related parameters was often exclusively documented in the area of infant cry diagnostics by eliciting pain-induced cries^61–67^. For many years, this approach hindered the recognition of the melody repertoire typical of spontaneous natural crying, which sets human infants apart from other primates^68^.

In earlier work and, remarkably even more recently, infant crying has been viewed as essentially stereotypic, similar to primate calls^69–71^. This view has since been refuted^35,44,45,49,72–78^. The mitigated, melodic cries of human infants are at some level, similar to simple musical melodies (“glissandi smoothly slurred or swept over a certain frequency interval”^17^; p.643) and could provide raw material for prosodic constituents of later language^17,37,58^. The same is true for prosodic properties of non-cry vocalisations.

The Melody Development Model (MD-Model), initially proposed by Wermke and Mende^45^ postulated that early vocal development is reflected by a sequence from simple to complex melody contours that span across infant crying and non-cry vocalisations providing important building blocks for prosody and language development. Furthermore, this complexity pattern is assumed to be regular, uni-directional for each vocalisation type, and universal in nature (ibid.). Melodic complexity is exhibited as an increase in the number of arc-like substructures of fo contour (melody arcs) for the combinatorial assembly of multiple-arc (complex) melodies of cry and non-cry vocalisations during the first months of life^17,45,72,77^.

**Figure 1:**
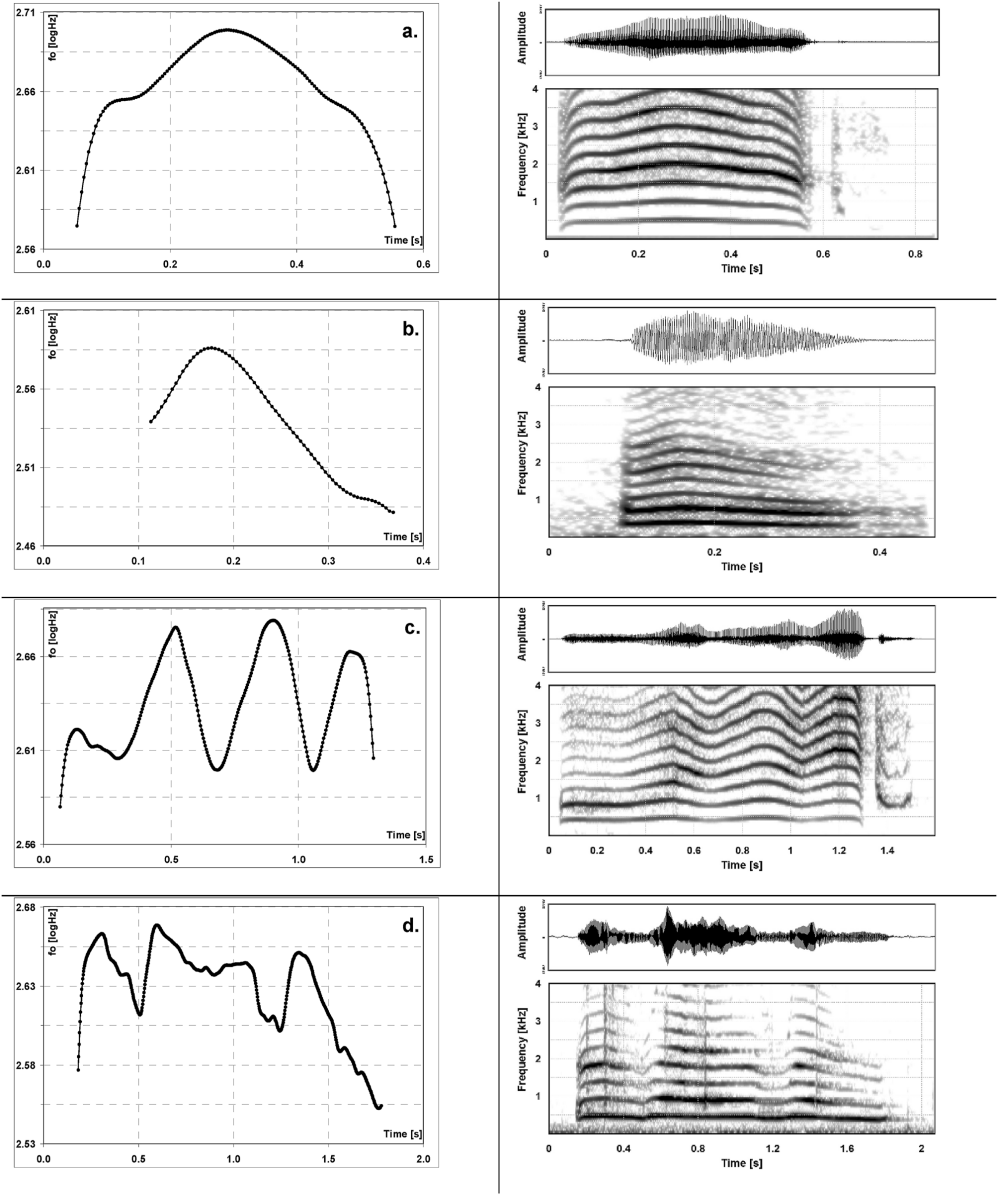
(**a**–**d**) Melody (fundamental frequency—fo) contour diagrams (left) exemplifying simple (a: cry, b: non-cry) and complex (c: cry, d: non-cry) patterns together with the corresponding frequency spectra (time representation above) of the sounds (up to 4 kHz). The frequency scale of the melody diagrams is logarithmic, and the diagram grid marks semitone (fo) vs. time distances. Note that spectra a. and c. also display the inspiratory noise following the vocalisation. For the original audio files of these vocalisations see Supplementary Information.

While newborn infant crying and early non-cry vocalisations (e.g., vocants as defined by Martin 1981^43^) show a melody contour that is still rather simple i.e. single-arc-like (see Fig. 1a,b), melody becomes more complex, i.e. multiple-arc-like with increasing age (see Fig. 1c,d). By the second to third month of life and depending on age-specific factors like individual fitness^79^ or sex hormone concentration during mini-puberty^73,80,81^ or the surrounding language^75,82,83^, melody structure in both vocalisation types becomes more complex, i.e. multiple-arc-like with increasing age.

To date, there is a small number of studies that have investigated melody development in infant vocalisation and few that have focused on melody development during the first six months of life^13,42,45,59^. The seminal paper by Kent and Murray (1982)^42^ examined the non-cry vocalisations in 3, 6 and 9-month-old infants during vocal interaction and play situations. Based on visual inspection of frequency spectrograms, they analysed “simple” and “complex” fo shapes to characterize intonation patterns produced by these age groups. Only 11% of multiple-arc (“complex”) patterns were observed at the age of 3 months (ibid., Table II p. 358). By 6 months, the number of complex melody contours had increased to 22% but reduced to 10% by 9 months. This may point to a developmental course of first increasing-then-decreasing production of complex melody contours in infant non-cry vocalisation between the age of 3 and 9 months. A longitudinal study of “speech quality” (segmental sound quality: vocalic versus syllabic vocalisations) and melodic complexity (suprasegmental prosodic features) in the non-cry vocalisations of infants between 2 and 6 months was reported by Hsu et al. (2000)^59^. However, melody contour was coded only qualitatively based on perceptual impressions. The researchers found a curvilinear trend that seemed to parallel the pattern reported by Kent and Murray (1982)^42^ of increasing then decreasing production of complex melodies in non-cry vocalisations. However, the decline in melody complexity was found at an earlier age (i.e., beginning at five months of age) compared to Kent and Murray (ibid.). The most comprehensive report on melody complexity development in infant crying was provided by Wermke and Mende^45^. The spontaneous cries of 270 infants were analysed for melodic complexity across the first five months of life. The authors described three developmental phases of cry melody, (1) birth to 8 weeks: initialization phase (increase of complex pattern from 30 to 52%), (2) 8 to 12 weeks: stabilization phase (no further increase) and (3) 12 to 18 weeks: modification phase (further increase up to a rate of about 65% cries exhibiting a complex melody; p. 34) during the third month of life was interpreted to be due to the emerging interaction of melody and resonance frequencies (resulting from vocal tract maturation). To the best of our knowledge, there are no further developmental studies of the melody features of infant vocalisations over the first six months of life, especially those capturing both cry and non-cry vocalisations.

To establish a more comprehensive model of early vocal development and gain a better understanding of early language (prosody) acquisition, we sought to investigate whether there is a developmental pattern of the production of complex melodies across the range of vocalisations produced during the first six months of life (i.e., encompassing cry and non-cry vocalisation types). The aim of the study was to perform an objective developmental analysis of prosodic precursors in the form of melody in healthy infants between 1 and 180 days of life in their cry *and* non-cry vocalisations. Based on the MD-Model by Wermke and Mende^45^, we hypothesised that both cry and non-cry vocalisations produced by infants would show a characteristic developmental increase in complex (multiple-arc) melodies.

## Methods

### Study design

Longitudinal analysis of complex melody pattern development among healthy young infants, stratified by cry and non-cry vocalisations from the baby sound archive at the Center of Pre-Speech Development and Developmental Disorders (University of Wuerzburg).

### Participants and datasets

Healthy, term-born (≥ 37 gestational weeks) monolingual German infants from birth to the age of 180 days of life. Exclusion criteria were any kind of hearing disorder or developmental disorders over the observation period. The available database totalled 67,629 vocalisations from 277 infants; comprising of 56,537 spontaneous cry utterances from 227 infants (115 boys; 50.7%) recorded between 1 and 180 days of life, and 11,092 non-cry vocalisations (cooing/babbling sounds) from 50 infants (24 boys; 47.1%) recorded between 60 and 180 days. Only one infant appeared in both cry and non-cry data sets (for more details see Table S1 in Supplementary information).

### Procedure

The archive contains anonymized audio files (wav format) of the original recording sessions (sequences of cry and non-cry vocalisations) as well as all the individual sounds, which were previously manually segmented using a commercially available system (CSL 4500; KayPENTAX, USA). Here, we used all cry and non-cry sounds available in the archive from our participants. Original recordings were approved by the respective ethical boards (ethics committee of the Charité, Humboldt University Berlin and ethics committee of the medical faculty of the University Wuerzburg) and were carried out in accordance with relevant guidelines and regulations; informed consent signed by parents was given. Finally, all recordings were archived as anonymized data sets. Each parent had a minimum of a high school education and the monthly family income was reflective of a middle class standard of living.

Cry vocalisations (spontaneous, naturally occurring crying) were recorded under comparable conditions in a hospital (first week) and at home, respectively (e.g., before breastfeeding, relaxed, pain-free manner). Non-cry-vocalisations were recorded in infants’ homes during joyful mother–infant interactions. All vocalisations were spontaneously uttered by the infants and any elicitation of vocalising was avoided. The length of an individual recording session ranged from about 1 to 3 min (crying) and 1 to 30 min (non-cry vocalisations).

### Vocalisation analysis

A cry or non-cry vocalisation was defined as an utterance produced on the expiratory phase of a single respiratory cycle and identified acoustically as the onset and offset of acoustic energy in the waveform. Frequency spectrograms were automatically calculated for each vocalisation using the CSL 4500 (KayPENTAX, USA). Based on visual inspections of the spectrograms, phonatory noise phenomena and phenomena like sudden fo shifts or subharmonics were identified. These well-known features of young infants’ vocalisations^84–86^ are often caused by strong nonlinearities in the restoring forces resulting from an extremely large amplitude-to-length ratio of the very young infants’ vocal folds^84,85^. Based on audio-visual inspections of the spectrograms (cf. previous detailed description^86^), vocalisations containing broad regions of phonatory noise (e.g., creaky sounds) and/or subharmonics or a highly unstable pattern caused by sudden frequency shifts (register changes) or marked vibrato-like phenomena were excluded from melody pattern analysis. The fo and its course over time (melody) cannot be reliably determined in those signals. The same was true for most vocalisations shorter than 300 ms to avoid effects from vegetative noises and sounds with background noise (e.g., parent’s voice). All vocalisations without a well-defined melody structure were identified by audio-visual analyses and categorized as exhibiting “no pattern” and subsequently excluded from the analysis. This means that “no pattern” represented a fuzzy class of vocalisations without a clearly definable melody. The remaining vocalisations were assigned as either containing a “simple” or “complex” melody pattern (see *Melody Complexity Analysis*). Statistical analysis revealed that both cry and non-cry vocalisation types had a decreasing occurrence of “no pattern” over age, but occurrence was lower in the non-cry vocalisations and decreased quicker than that for the cries:

For cries, the median age of cries without a well-defined melody (‘no pattern’) was 51 days, Q1 = 25 days, Q3 = 79 days, range: 1–173 days. Looking at the change over age, using a multi-level linear regression, there was a significant decrease in this pattern with increasing age (*p* < 0.001), given by the equation:

1 $$ {\text{proportion of cries with `no pattern'}} = 0.2773632{-}\left( {0.0003154 \times {\text{days}}} \right). $$

For non-cry vocalisations, the median age of sounds without a well-defined melody (‘no pattern’) was 113 days, Q1 = 96 days, Q3 = 140 days, range: 60–180 days. Looking at the change over age, there was a significant decrease in this pattern with increasing age (*p* < 0.001), given by the equation:

2 $$ {\text{proportion of non-cry vocalisations with `no pattern'}} = 0.1651677{-}\left( {0.0006172 \times {\text{days}}} \right). $$

For determining the melody pattern, an automatic fo measurement (melody contour analysis) was required using PRAAT v. 6.0.3^87^. PRAAT uses an autocorrelation method for fo analysis^88^. A post-processing verification included removal of high-frequency modulation noise and artefacts. In cases of obvious fo-tracking problems of the automatic routine, fo determination was manually repeated using PRAAT. Using specially developed lab intern software, melody diagrams were drawn and a low-pass filter (Gaussian filtering) was applied with a cut-off frequency of about 40 Hz to eliminate high-frequency modulation noise and artifacts^44^. This time-consuming, but robust analysis method, applied to each individual vocalisation, guaranteed that the subsequent melody pattern analysis was based on correct fo contours.

### Melody complexity analysis

Melody complexity analysis was performed using specific in-lab software (CDAP, pw-project), which was implemented as a routine procedure at the Center for Pre-Speech Development and Developmental Disorders. Using the fo data calculated with PRAAT, the CDAP software allows for flexible drawings of melody diagrams and quantitative melody contour analysis. To classify melodies in simple versus complex pattern, for each sound melody the number of single arcs was identified (cf. detailed description in Supplementary information). A melody arc was defined as being longer than 150 ms and as exhibiting a frequency amplitude (FM-amplitude) of at least three (cry) or two (non-cry) semitones^17^. In agreement with preceding studies^17,44,45,58^, a complex melody structure was defined as exhibiting ≥ two arcs and/or intra-melodic breaks between arcs by glottal oscillatory pauses or marked laryngeal constrictions that generate rhythmical variations of the acoustic Gestalt. Examples for rhythmic variations of complex (multiple-arc melodies) are available in several previous publications^37,45,58,89^.

Based on these objective criteria, all cry melodies were analysed and subdivided into those with only a simple (single-arc) melody (Fig. 1a,b), those with a complex (multiple-arc) melody (Fig. 1c,d), while those having “no pattern” had already been excluded during pre-processing (the excluded samples, see paragraph Vocalization Analysis).

### Statistical analysis

**Figure 2:**
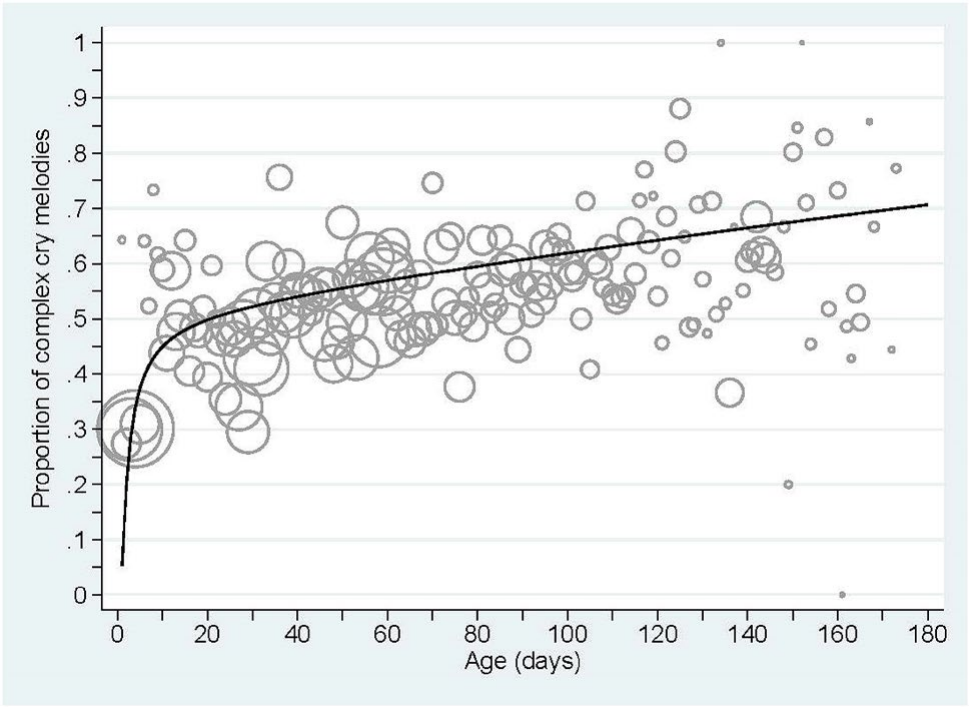
Bubble plot of the proportion of complex melodies in cries over age together with the mean prediction line derived from the final multi-level mixed effects logistic regression model. Note: the bubble size gives the relative number. Here, the median bubble size represented n = 197, with Q1 = 88, Q3 = 331 and range: 2–1804.

**Figure 3:**
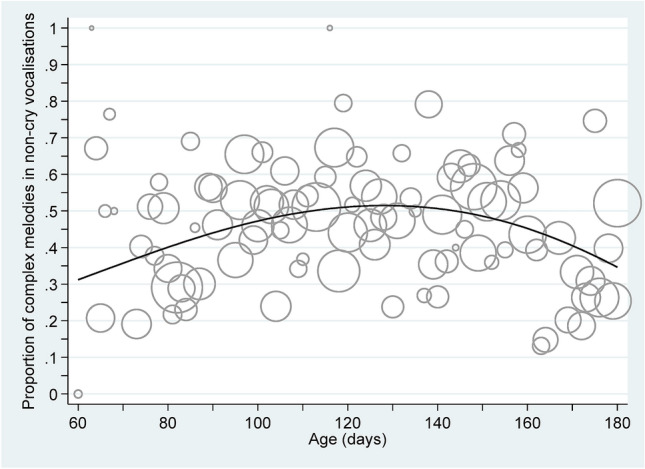
Bubble plot of the proportion of complex melodies in non-cry vocalisations over age together with the mean prediction line derived from the final multi-level mixed effects logistic regression model. Note: the bubble size gives the relative number. Here, the median bubble size represented n = 102, with Q1 = 45, Q3 = 143 and range: 2–347.

The reporting of analyses was informed by the Strengthening the Reporting of Observational Studies in Epidemiology (STROBE) guidelines; an international, collaborative initiative of epidemiologists, methodologists, statisticians, researchers and journal editors involved in the conduct and dissemination of observational studies (see: https://www.strobe-statement.org/)^90^. Analyses were stratified by melodies of cry and non-cry vocalisations. Summary statistics of infant’s sex, age and vocalisation signals were recorded, and the overall signal classification distributions were reported. Bubble plots (Figs. 2, 3) were then drawn, charting the proportion of vocalisations with complex melodies recorded by age (measured in days). Here, the bubble area reflected the relative numbers of vocalisations recorded at that age but ignored the nested nature of the data. The binary data (simple vs. complex melody pattern) were then analysed using fractional polynomial multi-level mixed effects logistic regression models, with unstructured covariance terms. Such models demonstrate flexibility and efficiency in modelling longitudinal developmental data, account for the hierarchical dependences associated with serial vocalisation data measurements nested within children over time, and minimise undesirable artifacts including edge effects and waves^91,92^. That is, results from polynomial regression models have a propensity to produce artefacts in higher order fitted curves—such as abrupt changes near the variable extremes, leading to unrealistic predictive data patterns^93^. Despite their flexibility and utility, fractional polynomial multi-level mixed effects logistic regression models have been rarely applied to non-Gaussian dependent variables^94^. Random intercept multi-level mixed effects logistic regression models were first investigated to determine the number of age related terms and their power function. Consistent with the recommendations of Royston and Sauerbrei (2008)^95^ degree-2 fractional polynomial powers of infant age were considered from the set (− 2; − 1; − 0.5; 0; 0.5; 1; 2; 3). The best models were then selected by minimising the deviance statistic, and the Χ^2^ test used to investigate deviance differences between models. These best fractional polynomial models defined the functional relationship of age (measured in days) to the binary complexity data for pursuant investigations. Once specified, both random intercept and random coefficient models were next investigated, and the Bayesian Information Criterion (BIC) used to select between these competing models^96^. The BIC rewards for goodness-of-fit to the data but penalises for model complexity, with the preferred model balancing these opposing demands and yielding the lowest BIC statistic. A sex difference was then tested, treating sex as a fixed effect. All statistical analyses and associated graphs were performed using Stata SE version 16.0 (StataCorp, College Station, TX, USA), and α = 0.05 defined significance.

## Results

### Regression analysis

#### Cry vocalisations

the mean recorded infant age of vocalisations was 56 days (SD = 38 days, range: 1, 173 days), with 21,789 (38.5%) signals defined to complex, 20,917 (37.0%) as simple, and 13,831 (24.5%) had ‘no pattern’ (and were subsequently set to missing). Overall, 226 infants had at least one recorded spontaneous cry utterances classified as simple or complex, with an average of 189 such recordings (range: 1, 910). Figure 2 presents a bubble plot of the crude proportion of complex cries (using the number of simple and complex cry signals as the denominator) by infant age (in days), without accounting for the serial nature of cries nested within children. A non-linear mean pattern is apparent within Fig. 2.

Fitting fractional polynomial multi-level mixed effects logistic regression models, the best model containing two powers of age, namely age^−1^ and age^1^ was significantly better than the best model containing one power of age (*p* < 0.001) or the model with a linear function of age (*p* < 0.001). This preferred random intercept model resulted in a BIC = 55,597. Extending the multi-level mixed effects logistic regression model to include both random intercept and random slopes for children yielded BIC = 54,815, a value superior to the random intercept only model. In this model, the fixed effects components were given by:

3 $$ {\text{logit}}\left( \Pi \right) = 0.0250{-}3.189/{\text{age}} + 4.711{\text{E}} - 03 \times {\text{age}} $$

where Π is the predicted binary response, and value 1 indicates complex cries while 0 indicates simple cries. This fixed effects function is also drawn on Fig. 2. Each of the fixed effects age terms were statistically significant (both *p* < 0.001); as were the random effects terms, with variability given by: constant SD = 1.13 (95% CI: 0.98, 1.30); age^−1^ SD = 5.94 (95% CI: 5.07, 6.96); age^1^ SD = 0.013 (95% CI: 0.011, 0.015); corr(age^−1^, age) = 0.56 (95% CI: 0.41, 0.72); corr(constant, age^−1^) = − 0.55 (95% CI: − 0.69, − 0.40); and, corr(constant, age) = − 0.82 (95% CI: − 0.89, − 0.75). No sex difference was identified (*p* = 0.13).

#### Non-cry vocalisations

the mean recorded infant age of vocalisations was 125 days (SD = 32 days, range: 60, 180 days), with 4527 (40.8%) signals defined to complex, 5428 (48.9%) as simple, and 1137 (10.3%) as having ‘no pattern’ (and were subsequently set to missing). Simple or complex cooing/babbling sounds were recorded from 50 infants; with an average of 199 such sounds (range: 1, 1332 sounds). Figure 3 presents a bubble plot of the crude proportion of complex signals (using the number of simple and complex melodies as the denominator) by infant age (in days), without accounting for the serial melody vocalisations nested within children. Again, a non-linear mean pattern is suggested within this figure.

This non-linear pattern was confirmed by the application of fractional polynomial multi-level mixed effects logistic regression models. The best model contained two powers of age, namely age^2^ and ln(age) × age^2^, and was significantly better than the best models containing one power (*p* < 0.001) or a linear function of age (*p* < 0.001). This preferred random intercept model yielded a BIC = 13,235. Extending the multi-level mixed effects logistic regression model to also include random slopes for children, the algorithm failed to converge when both age^2^ and ln(age) × age^2^ terms were simultaneously considered. However, the model including a random intercept and a random slope component for age^2^ yielded BIC = 13,040, a value superior to the random intercept only model. In this superior model, the fixed effects components were given by:

4 $$ {\text{logit}}\left( \Pi \right) = - 1.823 + 1.168{\text{E}} - 03 \times {\text{age}}^{2} {-}2.207{\text{E}} - 04 \times {\text{ln}}\left( {{\text{age}}} \right) \times {\text{age}}^{2} $$

where Π is the predicted binary response, and value 1 indicates complex melodies whereas the value 0 indicates simple melodies. These fixed effects function is superimposed on Fig. 3 as the solid line. The fixed effects for the age terms were statistically significant (both *p* < 0.001); as were the children’s random effects terms, with variability given by: constant SD = 1.066 (95% CI: 0.725, 1.568); age^2^ SD = 5.177E−05 (95% CI: 3.435E−05, 7.804E−05); and, corr(constant, age^2^) = − 0.813 (95% CI: − 0.999, − 0.614). Again, no differences between boys and girls was identified (*p* = 0.64).

## Discussion

Based on longitudinal analysis of melody structure (simple vs. complex pattern), we found that human infants acquire a repertoire of complex vocal melodies over the first six months of life, with rapid gains early on. Particularly impressive was the rapid increase of the prediction curve for melody complexity development derived from the final multi-level mixed effects logistic regression model in crying (Fig. 2). This behaviour demonstrates that human infants natural spontaneous crying (in contrast to pain cries) is much more than a simple alarm signal. That is, it seems unlikely and highly ineffective for a simple alarm signal to be characterized by rapid changes in the pattern and occurrence of complex melody.

The curve predicts a cry repertoire with about 53% of the vocalisations exhibiting a complex melody at the end of the first month and a further increase thereafter. This rapid gain requires mature functioning of neuro-physiological mechanisms underlying melody production, which corresponds to the fast brain growth at this early age^97,98^. For example, Holland et al.^97^ found that brain development is most rapid during the neonatal period with 64% of whole brain growth occurring within the first 90 days” (ibid. p. 6).

The early occurrence of complex cry melodies would further suggest a ‘preparatory’ intrauterine development. The muscles of the larynx are an integral part of the respiratory system. Like other respiratory muscles, they undergo considerable use prior to birth^99^. From birth, newborns are capable of a highly developed laryngeal-respiratory control that serves breathing and phonation^41,49,56,57^. This aptitude facilitates survival, but at the same time enables the newborn to play with his laryngeal options for vocalising and hence, to quickly acquire complex melody patterns.

Intrauterine preparation also involves perceptive components. Indeed, there is evidence that late-term fetuses demonstrate the ability to discriminate their native language, to which they were exposed in utero, from an unknown language^100,101^. These findings suggest that fetuses encode suprasegmental characteristics of speech (melody, rhythm) rather than segmental features, which is due to segmental information being filtered out by tissue and fluid before it reaches the fetus. Prenatal sensitivity to prosodic features was demonstrated in newborns, who exhibited a preference for a low-pass filtered maternal voice and language (focused on melody) in contrast to natural voice during their first days of life^5^.

The non-cry vocalisations produced by the infants were found to parallel a similar developmental path observed earlier for cry vocalisations. Specifically, the model curve for the non-cry vocalisations initially also shows a low percentage of complex melodies (about 30%) and a fast increase in their share of complex melodies over the next 2.5 months up to about 50% (Fig. 3). This vocal development is consistent with the course of brain development and reported perceptive performances of infants at this age^102^. From both, a phylogenetic and ontogenetic perspective on spoken language evolution, it seems essential that the rapid gain in producing crying characterized by complex melodies occurs long before supralaryngeal maturation (vocal tract maturation) allows for pre-articulatory activities in sound production.

Our observation that melody development in both cry and non-cry vocalisations is characterized by an increase in complexity points to a similar strategy in the acquisition and rehearsal of prosodic building blocks as postulated by the MD-Model, introduced by Wermke and Mende^45^. It emphasizes spontaneous crying being as important as non-cry vocalizations (cooing, babbling) during the developmental process. In contrast to past simplistic suggestions that human infant crying is a fixed and monotonous reflex response comparable to animal vocalizations^71^, we see a developmental progression of vocal growth. Our study further elucidates the prominence of melody variation in cry development. The early occurrence of complex melodies also confirms a previous report suggesting that by approximately two months of age, a majority of spontaneous cry vocalisations should contain complex melodies. Otherwise, the infant may be at risk for an early language impairment^79,89^. This hypothesis is supported by a recent study of Francois et al. (2017)^103^. The authors demonstrated that neonatal brain responses for sung streams predicted expressive vocabulary at 18 months. These findings further corroborate the importance of melody production and perception for language development long before “speech-like” vocalisations emerge.

There is major agreement between scientists of several disciplines, that the first universal steps taken by an infant on his way to spoken language include melodic-rhythmic aptitudes, with respect to both perceptive and productive performances. A systematic increase in melody complexity in cry and non-cry vocalisations, as demonstrated here, provides the raw material for later language prosody. This takes place to an extent that has been often underestimated in spontaneous crying and is continued in non-cry vocalisations. Consequently, infants’ melodic sound characteristics, i.e. early building blocks of prosody, are crucial in order to characterize their path to spoken language in the first months of life.

While the model curve of complex melodies among the cry repertoire did not decline at the end of the observation period, occurrence of complex melodies in non-cry vocalisations slightly decreased from about 4.5 to 5 months. This is most likely due to the new constituents of non-cry vocalisations that emerged to interact with the overall melodic contour, namely vowel-like (vocants) and consonant-like elements (closants). During the process of spoken language acquisition, the human infant must modify his laryngeally produced melodies repeatedly and tune them to the resonance frequencies of a vocal tract that continues to grow and change^42,44^. The tuning in non-cry vocalisations is additionally challenged by an increasing articulatory activity required to produce syllabic combinations in babbling from about five to six months on. This new developmental period requires a temporary “regression” in melody development to establish vocal development on a higher hierarchical level^17^. Thereafter, the infant begins to intentionally imitate intonation patterns of the surrounding language(s) in consonant–vowel syllable sequences in babbling^17,44,45^. The identification of primitive precursors of later articulatory speech elements (closants, constrictions), observed in early cry and non-cry vocalisations^49^, show the close interaction of suprasegmental and segmental phonatory activity by the infant at an early age. A logical next step in our line of research is to examine the possible interaction between vocant and closant articulations and melodic complexity.

The study provides the first statistical model to demonstrate a systematic melody development in cry and non-cry vocalisations of infants. Our data revealed a strong developmental continuity in spontaneous crying with respect to melody complexity across the first 180 days. Additionally, there was a continuous increase in complex melodies in non-cry development with a slight reduction occurring at approximately 140 days. Recognition of this developmental process will considerably improve not only our understanding of early preparatory processes for spoken language acquisition, but most importantly also allow for the creation of clinically robust risk markers for developmental language disorders. This is the crucial prerequisite to enable us to develop innovative therapies for infants at-risk for developing language disorders. This developmental model could help to better understand why the human infant acquires so quickly and seemingly effortlessly such a complex faculty as language.

## Supplementary Information

Supplementary Information.

Supplementary Audio 1.

Supplementary Audio 2.

Supplementary Audio 3.

Supplementary Audio 4.

## Data Availability

Because the participants did not give explicit written consent that their data can be made publicly available, data will not be shared.
