# Supplementary material for: Melody complexity of infants’ cry and non-cry vocalisations increases across the first six months
Source: Sci Rep. 2021 Feb 18;11:4137. doi: 10.1038/s41598-021-83564-8 (PMC7893022; doi:10.1038/s41598-021-83564-8)
Supplement: Supplementary file 1 — Supplementary Information. [file 41598_2021_83564_MOESM1_ESM.docx]

**Supplementary information**

**Melody complexity of infants’ cry and non-cry vocalisations increases across the first six months**

**K. Wermke, Michael P. Robb & Philip J. Schluter**

- **Table S1: Participants and their recordings**
- **Sound files of the four vocalisation examples (cf. their displayed melody patterns in Figure 1a-d of the main article).**
- **Figures SF1, SF2 displaying examples of sounds containing complex melodies**

**Table S1: Number of infants that had recordings included per months (different age in days) across the observation time**

| **Cry** | **Recordings in all 6 monthly periods** | **Recordings in 3 to5 out of 6 monthly periods** | **Recordings in less than 3 out of 6 monthly periods** |
| --- | --- | --- | --- |
| Number of infants | 16 | 97 | 113 |
|  |  |  |  |
| **Non-cry** | **Recordings in all 4 months** | **Recordings in 3 out of 4 months** | **Recordings in less than 3 out of 4 months** |
| Number of infants | 9 | 2 | 39 |

**Audio files:**

**Sa_cry_simple melody.wav**: sound file – cry vocalisation exhibiting a simple, one-arc melody pattern (age: 32d)

**Sb_non-cry simple melody.wav**: sound file – non-cry vocalisation exhibiting a simple, one-arc melody pattern (babbling; age: 116d)

**Sc_cry_complex melody.wav:** sound file – cry vocalisation exhibiting a complex, triple-arc melody pattern (age: 88d)

**Sd_non-cry_complex melody.wav**: sound file – non-cry vocalisation exhibiting a complex, triple-arc melody pattern (babbling; age: 156d)

| 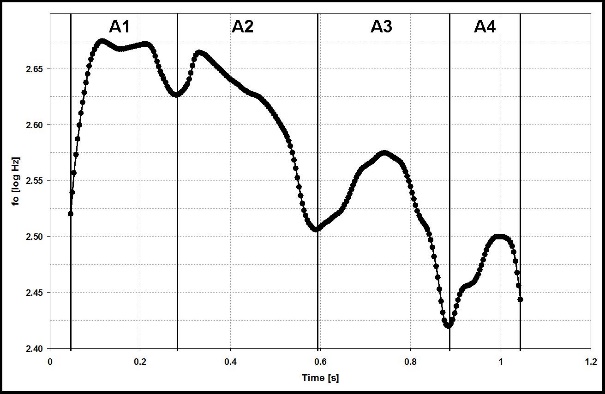 | 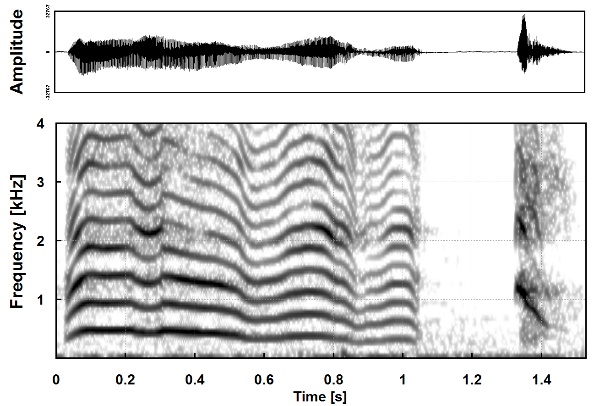 |
| --- | --- |
| 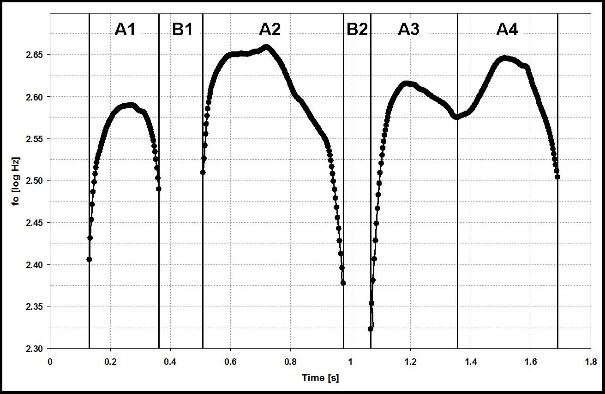 | 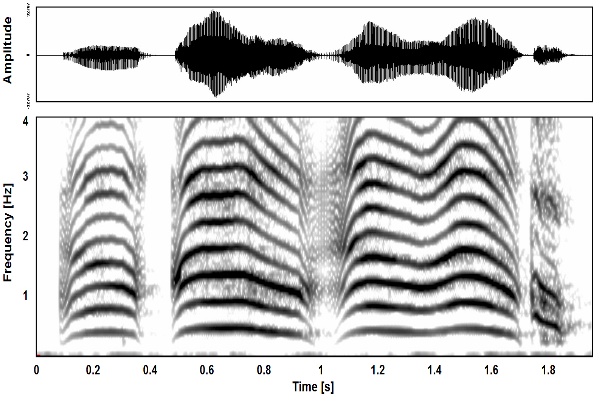 |

| **Figures SF1, SF2 displaying examples of sounds containing complex melodies and melody arc identification (A1 – first arc, A2 second arc…B1 – oscillatory break..).** Melody diagrams (left) and (right) time representation and frequency spectrogram of vocalisations containing a complex melody. In both examples, the inspiratory noise following the single cry vocalisation is displayed in the spectra. The sound example below contains two oscillatory breaks (laryngeal constrictions). |
| --- |

The lab-intern software to objectively evaluate melody complexity is not yet ready for public use, but certainly will be in future.
